# Astaxanthin Decreases Spatial Memory and Glutamate Transport Impairment Induced by Fluoride

**DOI:** 10.22037/ijpr.2021.114919.15107

**Published:** 2021

**Authors:** Farzaneh Mirsaeed-Ghazi, Mohammad Sharifzadeh, Mohammad Reza Ashrafi-Kooshk, Saeed Karima, Sogol Meknatkhah, Gholamhossein Riazi, Farzad Mokhtari

**Affiliations:** a *Institute of Biochemistry and Biophysics, University of Tehran, Tehran, Iran.*; b *Department of Toxicology and Pharmacology, Faculty of Pharmacy, Tehran University of Medical Sciences, Tehran, Iran. *; c *Department of Clinical Biochemistry, School of Medicine, Shahid Beheshti University of Medical Sciences, Tehran, Iran. *; d *Behbalin Co. Ltd. Clinical Trials, Tehran, Iran.*

**Keywords:** Fluoride, Astaxanthin, Glutamate, ROS, Learning and Memory

## Abstract

Excessive exposure to the sources of fluoride in drinking water, oral care products, and food is a widespread problem. Fluoride is associated with impairment in child intelligence development. It causes DNA damage, oxidative stress, and mitochondrial dysfunction, mainly due to the production of reactive oxygen species** (**ROS). It has been postulated that the use of antioxidants such as astaxanthin, may alleviate fluoride’s adverse effects. This study assessed the effects of fluoride on cellular ROS content and rat’s learning and memory ability and investigated the protective potency of astaxanthin with emphasis on the role of glutamate using the Morris Water Maze test, glutamate concentration determination, and western blot techniques. The fluoride treatment of cells results in an increment of cellular ROS, whereas astaxanthin inhibits lipid peroxidation. Fluoride significantly decreases the cellular glutamate uptake and glutamate transporter, protein level, possibly due to the disruption of mitochondrial energy metabolism and defect of the transporter recycle, respectively. The *in-vivo* study indicated that the treatment of rats with fluoride led to a loss of learning, while astaxanthin improved memory dysfunction. Measurement of ROS and glutamate levels of rat brain hippocampus showed that fluoride increased the ROS but decreased the glutamate. On the other hand, the utilization of astaxanthin decreased the brain ROS content and increased the glutamate level. It seems that fluoride disrupts the normal function of neurons via increment of ROS production and decrement of glutamate level, whereas astaxanthin has neuroprotective potency due to the ROS scavenging ability.

## Introduction

A proper number of research studies have demonstrated the toxic effect of fluoride on human health (1, 2). The major source of fluoride for most people is water (3). Excessive exposure to fluoride sources in drinking water and fluoride-containing toothpaste and mouthwashes is predicted to change one’s metabolism (4, 5). Fluoride is associated with impairment in child development and retarded intelligence in children under extreme tooth fluoride therapy (6). Fluoride accumulates in soft tissues of the body, such as Nerves, kidneys, and the liver (7), and affects enzymatic activity (8). A major concern related to fluoride exposure is neurotoxicity or neuronal damage (9). Neurons exposed to sodium fluoride undergo DNA damage, oxidative stress, and mitochondrial dysfunction (10). 

Based on the electronegativity of fluoride ions, fluoride can cross the blood-brain barrier (BBB) and lead to memory and learning deficiencies (11-13). Substantial evidence declares that free radical production, distortion of energy metabolism, and lipid peroxidation are the common mechanisms of fluoride neurotoxicity in the central nervous system (CNS) (14-16). One side-effect of fluoride toxicity is the decrement of brain glutamate content, which has a fundamental role in memory formation. Alongside this, reports indicate that the changes in glutamate levels in the rat hippocampus can defect its learning and memory ability (17). Glutamic acid is the predominant excitatory neurotransmitter within the vertebrate CNS, which is involved in the process of cognition, learning, and memory (18). Glutamate exerts a signaling role by acting on glutamate receptors that are located on the cell surface. Therefore, extracellular glutamate concentration determines the stimulation of glutamate receptors. Excessive receptor activity causes excitotoxicity, leading to neuronal death. Since no enzymes are known to metabolize glutamate in the synaptic space, the only possible mechanism for glutamate elimination is its uptake through the glutamate transporters present in the membranes of neurons and adjacent astrocyte cells (19). Glutamate transporters, also called excitatory amino acid transporters (EAATs), remove the synaptically released glutamate from the extracellular space (20). Many studies have tried to connect the glutamate level with fluoride. There is no evidence as yet, however, for the effect of fluoride on the glutamate transporter. Hence, in this study, the impact of fluoride on cell-expressed EAAT2 was investigated using the western blot technique. 

Several approaches have been examined to minimize/eliminate the adverse effects of fluoride. Since the enhancing of reactive oxygen species (ROS) production is one of the major mechanisms of fluoride toxicity, it can be useful to utilize antioxidants (21). A natural carotenoid (3,30-dihydroxy-b,b-carotene-4,40-dione), astaxanthin (ATX) is a red-orange pigment found in crustaceans, salmonids, shrimps, and red yeast (22). ATX has been proven to have diverse biological activities, including anti-inflammatory (23), anti-apoptotic (24), and anti-carcinogenic effects (25). Recent studies have investigated the neuroprotective effects of ATX by crossing the brain-blood barrier (26). As a strong ROS scavenger, ATX effectively reduces oxidative stress in different cell and animal models (27). Lately, the potent antioxidant ATX has been shown to alleviate the damages caused by glutamate excitotoxicity to PC12 cells (28). 

Here, it is hypothesized that ATX treatment can protect astrocyte cells from death against fluoride side effects. As fluoride can cause glutamate deficiency in astrocytes, the question of whether ATX can effectively issue the fluoride neurotoxicity or not was investigated. To verify this, the Morris Water Maze (MWM) test, ROS measurement, glutamate concentration determination, and western blot were carried out.

## Experimental

*Cellular assays*

*Cell culture*

human astrocytes, which were isolated earlier from the hypothalamus and cerebral cortex of two human fetuses (obtained from Bon Yakhteh Laboratory in Tehran), were cultured in Dulbecco’s Modified Eagle’s medium (DMEM, Gibco, UK) supplemented with 10% fetal bovine serum (FBS, Gibco, UK) and 1% penicillin-streptomycin (Gibco, UK) at 37 °C in 5% CO2 atmosphere.

*Measurement of cell viability using the MTT assay*

Primary fetal human astrocytes were seeded in a 96-well plate (10,000 cells per well) in complete DMEM and were incubated overnight. They were then exposed to various concentrations (10, 30, 50, 70 and 100 μΜ) of ATX, a gift from Dr. M. R. Rashidi (Tabriz University of Medical Sciences), solved in dimethyl sulfoxide (DMSO, C99.9 %) for 12 and 24 h and exposed to various concentrations (0,1, 2, 4 and 6 mM) of sodium fluoride (NaF, C99 %, Sigma-Aldrich, USA) solved in water for 6, 12, 24, and 48 h. After incubation, 20 µL of 3-(4,5-dimethylthiazol-2-yl)-2,5-diphenyltetrazolium bromide (MTT, a tetrazole; SigmaAldrich) solution (5 mg/mL in phosphate-buffered saline [PBS]) was added to each well and incubated for 4 h at 37 °C.

After discarding the solution, 100 µL of DMSO was added, and the plates were shaken for 15 min. The absorbance of each sample was read at 570 nm using a microplate reader. The outcomes were affirmed as a percentage of cell viability with respect to untreated control cells (as 100%).

*Measurement of intracellular ROS*

Cellular generation of ROS in astrocyte cells was measured using the fluorescent probe 2’,7’-dichlorodihydrofluorescein diacetate (DCFH-DA, Sigma-Aldrich, USA). After drug treatment in our assay, cells were incubated with 10 µM DCFH-DA for 30 min at 37 °C in dark, washed twice in PBS, and analyzed by a Cary Eclipse Spectrofluorometer (Varian Inc.) with excitation and emission wavelengths of 485 and 530 nm, respectively. 

*Measurement of cell glutamate uptake*

Extracellular glutamate levels were measured by a fluorometric method using a glutamate assay kit (ab138883). After treatment, 100 µM glutamic acid (Sigma-Aldrich, USA) was added to each well and incubated for 15 min. Fifty microliters of supernatants were transferred into 96-well microplates, mixed with 50 µL substrate mixture, and incubated in RT. Detection of fluorescence reaction was determined using an automated microplate reader at a wavelength of Ex/Em = 540/590 nm. Glutamate concentrations were subsequently estimated from the standard curve using known l-glutamate amounts. Glutamate was measured in cell lysates, showing quantity (nmol) per mg of the extracted protein. The concentration of protein was measured by the Bradford method (29), using bovine serum albumin (BSA) as the standard.

*SDS-PAGE and western blot analysis*

After treatments, cells were lysed in lysis buffer (Tris 20 mM, NaCl 150 mM, EDTA 1 mM, sodium pyrophosphate 2.5 mM, NaF 20 mM, β-glycerophosphoric acid 1 mM, and sodium orthovanadate 1 mM). The western blotting analysis was carried out following the procedure described by Fatemi *et al. *(30). Equal amounts of protein were separated by SDS–PAGE and transferred to polyvinylidene difluoride (PVDF) membranes. The membranes were subsequently incubated with specific primary antibodies and then washed three times by Tris-buffered saline (TBS) containing 0.1% Tween-20. For detection, membranes were incubated with anti-rabbit IgG-horseradish-peroxidase and developed using an ECL plus kit (Amersham Bioscience, Buckinghamshire, UK), exposed to Kodak autoradiographic films. Densitometry analyses of bands were accomplished by the ImageJ application. 

*Animal assays*

*Animals*

Forty-two mature male Wistar rats weighing 100-120 g were used in this research. All animal experiments met the National Institutes of Health guidelines for the care and use of laboratory animals (NIH Publications No. 8023, revised 1978). Also, the ethical code was obtained from the Medical Ethics Committee of Tehran University of Medical Sciences. One week before the study, the rats were housed in standard conditions with a 12-h dark–12-h light cycle at 25 °C ± 2 °C and fed with a standard diet of pellets and clean tap water. Animal experiments were conducted under the regulations set by the Animal Care and Use Committee of Tehran University of Medical Sciences guidelines. Animals were assigned into six groups each containing seven rats and were treated as follows: Group 1 served as untreated controls (C). The rats in Group 2 (NaF) were given orally NaF dissolved in distilled water at a dose of 270 ppm for 4 weeks. Group 3 rats were subjected to gavage ATX dissolved in olive oil at a dose of 25 mg/kg bw/day for 2 weeks. Fluoride concentration was chosen based on cell culture experiments and published previous studies (31-34). The maximum concentration of fluoride that was used in the cell viability test was 6 mM which equals 250 ppm. On the other hand, the dose of astaxanthin was selected according to studies that surveyed its dietary supplementation and neuroprotective potency in rats (35-38). Group 4 (ATX+ NaF) rats were given NaF followed by ATX at the same mentioned dose. Group 5 (NaCl) rats were given orally NaCl dissolved in distilled water at a dose of 270 ppm for 4 weeks. Group 6 (sham) rats were given olive oil by gavage for 2 weeks.

*Bodyweight and water consumption measurement*

The body weights of rats at 1, 7, 14, 21, and 28 days and the volume of water consumed by each respective group were noted and analyzed.

*Morris Water Maze test (MWM)*

Learning and memory of all rat groups were examined after treatment, using the MWM test (39). The maze was a round black pool, 150 cm in diameter and 60 cm in height. The maze was filled with water to a depth of 40 cm at a temperature of about 23 °C. Four identically spaced places at the circumference of the pool divided the pool into four quadrants and were used as the beginning points. An escape platform of 10 cm diameter was located 2 cm beneath the surface of the water at a fixed position in the center of one of the quadrants. All rats participated in a daily session of four training trials for four consecutive days. Each rat was permitted to find the latent platform within 90 s. The time spent to detect the platform (Escape latency), the distance each rat swam to find the platform (Path length), and swimming speed (Velocity) were recorded. One day after acquisition, a probe test was performed to evaluate memory level by removing the platform (40).

*Measurement of intracellular ROS*

The intracellular generation of ROS was measured using DCFH-DA. Briefly, the hippocampus of rat brains was homogenized in PBS, incubated with 10 µM DCFH-DA for 30 min at 37 °C in dark, and analyzed by a Cary Eclipse Spectrofluorometer (Varian Inc.) with excitation and emission wavelengths of 485 and 530 nm, respectively. The data of the treated group are presented as a change in fluorescence as compared to control.

*Measurement of glutamate level*

Extracellular glutamate level was measured by a fluorometric method using a glutamate assay kit (ab138883). The brain tissue of the rats was harvested after treatment, and the hippocampus was removed and homogenized. Fifty microliters of supernatants were transferred into 96-well microplates, mixed with 50 µl substrate mixture, and incubated in RT. Fluorescence reaction was detected using an automated microplate reader at a wavelength of Ex/Em = 540/590 nm. Glutamate concentrations were subsequently estimated from the standard curve using known L-glutamate amounts.

*SDS-PAGE and western blot analysis*

The brain tissue of the rats was harvested. Subsequently, the hippocampus was dissected, homogenized, and then lysed in a lysis buffer (Tris 20 mM, NaCl 150 mM, EDTA 1 mM, sodium pyrophosphate 2.5 mM, NaF 20 mM, β-glycerophosphoric acid 1 mM, sodium orthovanadate 1 mM). The western blotting analysis was carried out following the method described by Fatemi *et al.* (30). Equal amounts of protein were separated by SDS–PAGE and transferred to polyvinylidene difluoride (PVDF) membranes. The membranes were subsequently incubated with specific primary antibodies and then washed three times with a Tris-buffered saline (TBS) containing 0.1% Tween-20. For detection, the membranes were incubated with anti-rabbit IgG-horseradish-peroxidase and developed using an ECL plus kit (Amersham Bioscience, Buckinghamshire, UK) exposed to Kodak autoradiographic films. Densitometry analyses of bands were accomplished using the ImageJ application. 

*Statistical Analysis*

All data are represented as the mean ± SD from three independent experiments, except for the animal studies. One-way ANOVA along with Tukey’s post-hoc test was performed using the GraphPad Prism software version 6.0 for Windows, GraphPad Software, La Jolla California USA, www.graphpad.com. Statistical significance was expressed as ^*^P < 0.05; ^**^P < 0.01; ^***^P < 0.001; ^****^P < 0.0001. Data were compared with the non-treated controls.

## Results and Discussion

*Cellular assay results*

*Effect of NaF exposure and ATX pretreatment on the viability of astrocyte cells*

**Figure 1:**
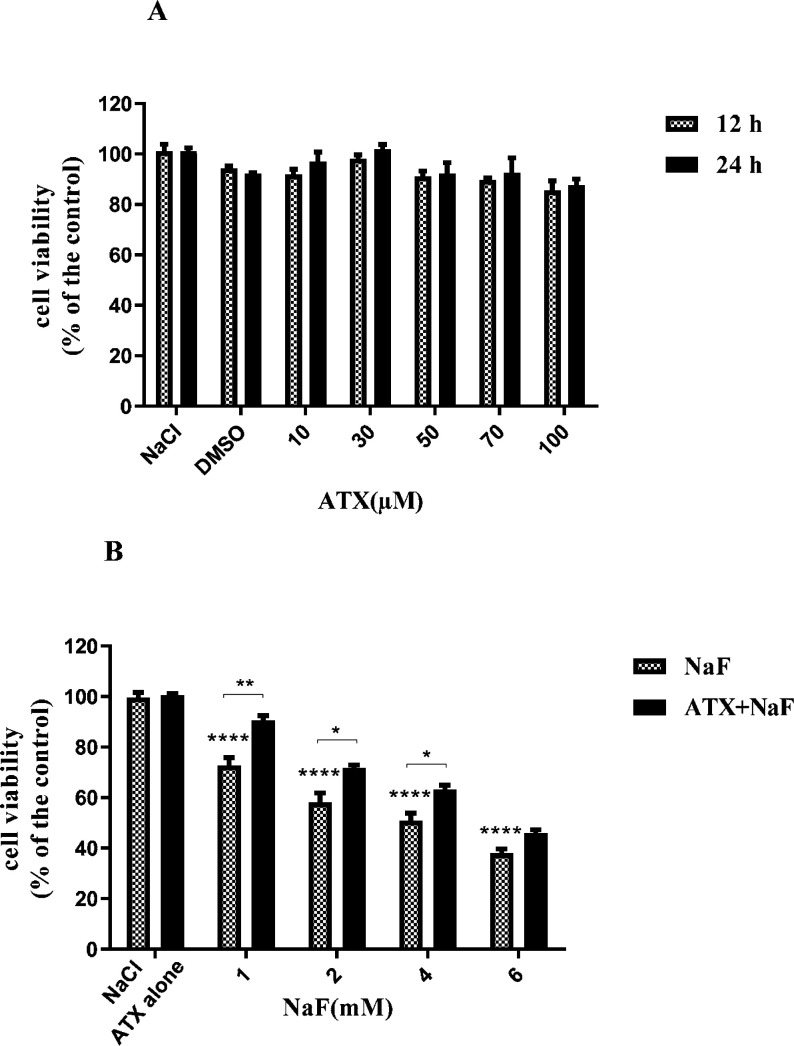
Effect of NaF exposure and ATX pretreatment on the viability of astrocyte cells. (A) Astrocyte cell viability was measured by MTT assay at 12 h and 24 h after treatment with different concentrations of ATX. (B) Cells were exposed to 1, 2, 4, and 6 mM NaF alone (NaF) and after pretreatment with 30 μM ATX (ATX+NaF). The NaCl, DMSO and ATX alone as controls were also shown

To evaluate the influence of ATX on the viability of astrocyte cells, we exposed cells to different concentrations of ATX (10-100 μΜ) so that the dosage of ATX applied in the subsequent experiments could be determined. ATX did not show remarkable cytotoxicity when it was incubated with astrocyte cells (Figure 1A) within 24 h. Also, to survey the effect of NaF on the viability of astrocyte cells, the cells were treated with different concentrations of NaF (1-6 mM) for 24 h. The results of MTT assays revealed that NaF lowered cell viability in a dose-dependent manner in comparison with the control group. However, pretreatment with 30μΜ of ATX for 12 h significantly increased cell viability (*P* < 0.05, Figure 1B). These findings indicate that ATX has a protective impact on astrocyte cells against NaF-induced cytotoxicity. These results are in line with previous reports, which indicate that NaF has cytotoxicity in the mM range (41, 42). ATX can transfer across the blood-brain barrier and has neuroprotective effects (34, 43).

*Effect of NaF exposure and ATX pretreatment on the production of intracellular ROS*

Shuhua *et al.* report that fluoride ions increase ROS *in-vitro*. Besides, Dose *et al.* have indicated that ATX has an antioxidant property (16, 44), whereas there is no evidence to suggest the effect of ATX on ROS induction by NaF. 

**Figure 2:**
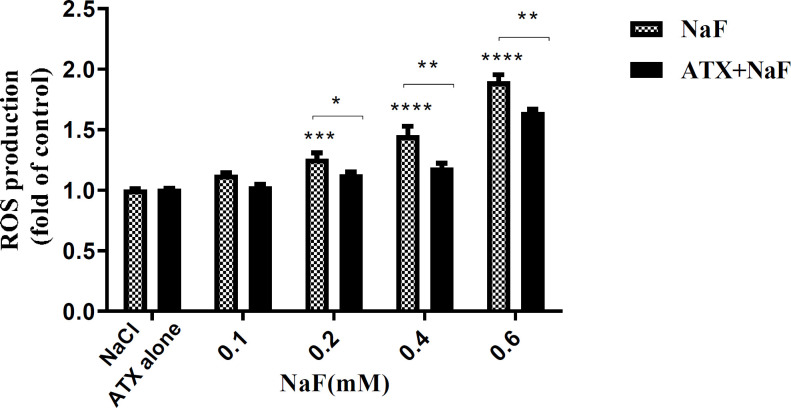
Effect of NaF exposure and ATX pretreatment on the production of ROS in astrocyte cells*.* Cells were exposed to 0.1, 0.2, 0.4, and 0.6 mM NaF alone (NaF) and after pretreatment with 30 μM ATX (ATX+NaF). The NaCl and ATX alone as controls were also shown. The results were expressed as a ratio to control value

Hence, the adverse effects of NaF on intracellular ROS and the protective potency of ATX were investigated by the DCFH-DA method. Accordingly, the cells were initially treated with 30 µM ATX for 12 h and subsequently with 0.1 - 0.6 mM NaF for 24 h. The fluorescence intensity of DCF-DA increased in the presence of ROS. As shown in Figure 2, ROS levels increased in NaF-treated astrocyte cells in a dose-dependent manner, while ATX pretreatment significantly suppressed intracellular ROS production compared with NaF alone. NaCl and ATX alone were used as controls, which indicates that they have no remarkable ROS induction and prevention ability, respectively. 0.6 mM of NaF increased the ROS content by 50% (*P *< 0.0001), whereas ATX pre-treatment reduced ROS production by 50% (*P* < 0.01). 

NaF may inhibit the complex IV of the respiratory chain, leading to increased production of superoxide radicals and thereby of hydroxide peroxide and peroxynitrite (45). However, the activities of antioxidant enzymes, such as Superoxide dismutase (SOD), Catalase (CAT), and Glutathione peroxidase (GPx), cannot prevent an increase in free radical formation due to (complex IV) inhibition. Therefore, there would be a rise in ROS, which may finally produce oxidations in membranes and the cell macromolecules (as seen by increased lipid peroxidation) and may underlie the reduced production of mitochondrial energy (ATP) (46). On the other hand, due to structural properties, ATX scavenges the produced ROS and activates SOD and catalase, which results in a decrement in the intracellular ROS level.

*Effect of NaF exposure and ATX pretreatment on cell glutamate uptake (in-vitro)*

Two major processes guided by astrocytes in the CNS are the uptake of glutamate from the extracellular space and glutamate release to neurons, which protect against glutamate excitotoxicity and strengthen neuronal firing, respectively (47). During inflammatory conditions (due to/lead to ROS production) in the CNS, astrocytes may lose one or both of these functions, resulting in the accumulation of extracellular glutamate and, eventually, excitotoxic neuronal death. This, in turn, worsens CNS inflammation (48). Therefore, the glutamate uptake activity of cultured astrocytes was evaluated by the clearance of L-glutamate from the extracellular space. Astrocyte cell cultures were pretreated with 30 µM ATX for 12 h and subsequently treated with 0.1-0.6 mM NaF for 24 h. Afterward, the assay was initiated by adding 100 µM L-glutamate and was measured after 15 min. In the presence of NaF, the residual extracellular L-glutamate was enhanced in a dose-dependent manner versus the controls.

**Figure 3:**
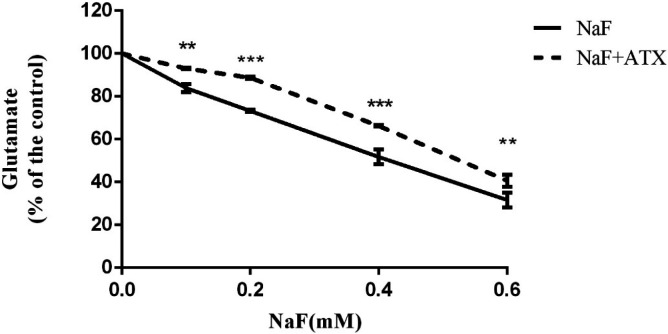
Effect of NaF exposure and ATX pretreatment on cell glutamate uptake*.* Cells were exposed to 0, 0.1, 0.2, 0.4, and 0.6 mM NaF alone (NaF) and after pretreatment with 30 μM ATX (ATX+NaF). The results were expressed as a percentage to control value

 On the other hand, in the ATX pretreated groups, the L-glutamate uptake improved in comparison with corresponding groups (*P* < 0.01, Figure 3). In the ATX-treated cultures, extracellular L-glutamate levels were reduced compared with the NaF-treated groups (*P* < 0.01) after 15 min of incubation (Figure 3). 

Based on outcomes, it is speculated that fluoride generates intracellular ROS, which affects glutamate uptake and cellular and mitochondrial membrane permeability. Fluoride-stimulated ROS can decrease the glutamate uptake directly upon inhibition by peroxynitrite and hydrogen peroxide and arachidonic acid produced from lipid peroxidation (49, 50), or indirectly by an increment in membrane permeability.

 Membrane lipids’ peroxidation causes a conformational change in phospholipids, leading to increased membrane permeability and, in turn, disrupted mitochondrial energy production (51, 52). The deficiency of mitochondrial energy metabolism (ATP production) results in the accumulation of intracellular sodium concentration due to the failure of Na^+^/K^+^ ATPase (53). Also, it has been reported that fluoride inhibits Na^+^/K^+^ ATPase by altering metal ions concentration (such as Mg2^+^ and Mn2^+^), enzyme activity (such as ENO1, PKC, and ALP), hormones (such as PTH and TSH), and enhancing cyclic nucleotides (such as cAMP and cGMP), Pi, and NO (53). Glutamate transporters are coupled to Na^+^/K^+^ ATPase (54). In the meantime, the weakness of the sodium gradient disrupts the function of the glutamate transporter (EAAT2/GLT-1) and causes a reduction in glutamate uptake. However, ATX has antioxidant properties and improves glutamate uptake by integrating into the lipid bilayer (therefore amending the mitochondrial membrane permeability) and preventing lipid peroxidation and ROS production (such as peroxynitrite and nitric oxide) (55).

*Effect of NaF exposure and ATX pretreatment on EAAT2 protein expression*

**Figure 4:**
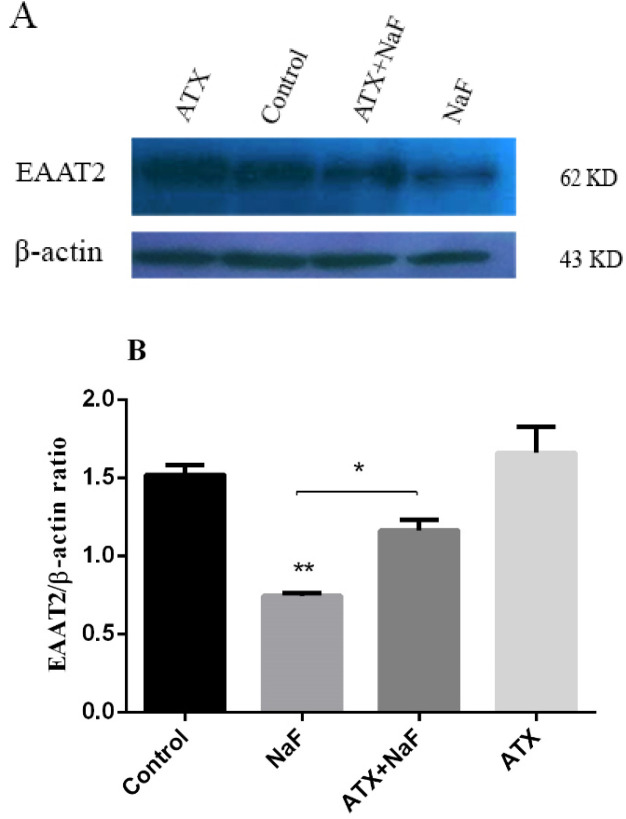
Effect of NaF exposure and ATX pretreatment on EAAT2 protein expression. (A) Western blot analysis of groups with exposure to 0.2 mM NaF alone (NaF) and after pretreatment with 30 μM ATX (ATX+NaF) was carried out after 24 h. Control and ATX groups were treated without NaF/ATX and with ATX alone, respectively. β-actin was used as a cell house-keeping protein. (B) The expression of EAAT2 was semi-quantitated as a ratio to β-actin

EAAT1 is expressed during a person’s development through childhood to adulthood, whereas EAAT2 is the main glutamate transporter in the adult brain, responsible for over 90% of the total glutamate uptake (56). Since EAAT2 is the major glutamate transporter in astrocytes, its presence and abundance were investigated by the western blot technique. Astrocyte cells were pretreated with ATX (30 μΜ) for 12 h and exposed to NaF (0.2 mM) for 24 h. The protein expression of glutamate transporter (EAAT2) was determined by western blot analysis. As shown in Figure 4, NaF suppressed EAAT2 expression compared with the control group (*P* < 0.01). Pretreatment with ATX increased EAAT2 expression compared with the NaF alone group (*P* < 0.05). The treatment of astrocyte cells with ATX increased EAAT2 expression. As mentioned above, the glutamate uptake was impaired in the presence of NaF, whereas pre-incubation with ATX initially restored the function.

 Reduced glutamate uptake can be due to the inhibition of the EAAT2 transporter, energy-lack dysfunction of the transporter, or a decrement of onboard membrane active transporter. The western blot results indicate that the abundance of the transporter in astrocytes decreases in NaF presence, whereas pretreatment with ATX increases the transporter significantly. Nonetheless, these results do not indicate that all transporters are active or that the inhibition of transporters does not occur. 

The cell surface turnover of the EAAT2 transporter is mediated via ubiquitination/deubiquitination catalyzed by PKC-mediated Nedd4-2 and UCH-L1, respectively (57). Ubiquitination of EAAT2 by Nedd4-2 which is activated by PKC leads to cell surface availability of transporter, whereas deubiquitination by UCH-L1 causes endocytosis and lysosomal degradation of the transporter. When no energy is available because of fluoride, the ATP-dependent function of PKC-mediated Nedd4-2 gets suppressed, whereby ubiquitination and exocytosis of the transporter reduce. However, deubiquitination by UCH-L1 does not rely on ATP and occurs as usual. It appears that the energy deficiency due to disruption in mitochondria function leads to a decrease in EAAT2 exposure, while the degradation of the transporter by deubiquitination occurs continuously. Besides, it has been demonstrated that ATX can well mitigate oxidative stress-induced under various pathological conditions, including poor diet or bad eating habits (58), infection, and inflammation, and, hence, prevent oxidative stress-induced mitochondrial dysfunction (59-61). Similarly, fluoride can cause a dysfunction in mitochondria through oxidative stress, which may inactivate the respiratory chain of mitochondria and change membrane permeability. ATX restores mitochondria’s performance by keeping its structural and functional integrity, which can inhibit the onset or progression of human diseases. Well-functioning mitochondria produces sufficient energy to be consumed in ubiquitination/deubiquitination of the transporter protein. 

*Animal assay results*

To investigate the adverse effects of NaF on rat brain and the protection potency of ATX, the animals were first gavaged with ATX (25 mg/kg bw/day) for 2 weeks and subsequently orally treated with NaF solution (270 ppm) for 4 weeks. After treatment, the total glutamate concentration of the hippocampus correlated with the results of the MWM behavioral test. Also, the total oxidant/antioxidant status and expression of GLT1 (equal with human EAAT2 transporter) were examined to confirm the *in-vitro* cell culture results by *in-vivo* outcomes. 

*Effect of NaF exposure and ATX pretreatment on water consumption and body weight of the treated animals*

**Figure 5:**
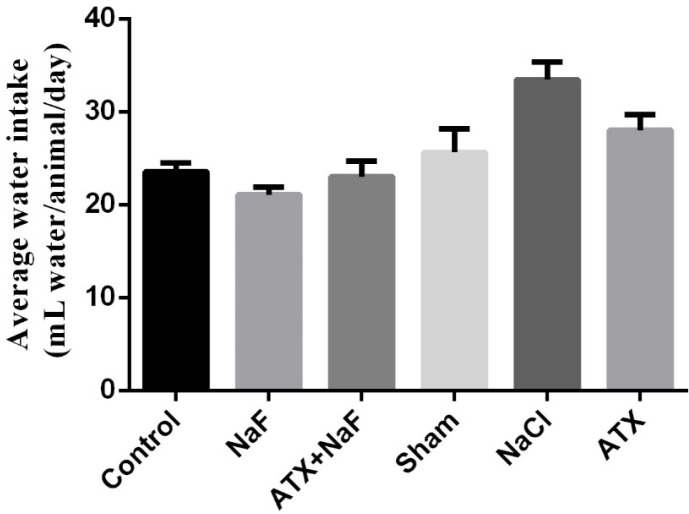
Effect of NaF exposure and ATX pretreatment on the water consumption of rats. The rats were treated with 270 ppm NaF alone (NaF), 25 mg/kg bw/day ATX pretreated (ATX+NaF), and 25 mg/kg bw/day ATX alone (ATX). The control group received no treatment, whereas sham and NaCl groups were treated with olive oil and NaCl solution, respectively

**Figure 6:**
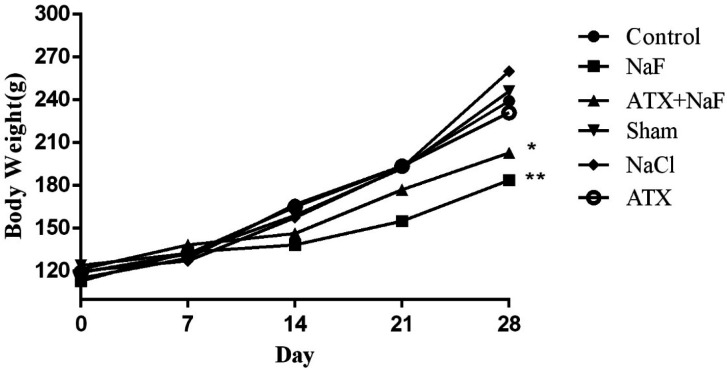
Effect of NaF exposure and ATX pretreatment on the body weight of rats. The rats were treated with 270 ppm NaF alone (NaF), 25 mg/kg bw/day ATX pretreated (ATX+NaF), and 25 mg/kg bw/day ATX alone (ATX). The control group received no treatment, whereas sham and NaCl groups were treated with olive oil and NaCl solution, respectively

To ensure that the rats in different groups have similar nutritional and physical characteristics, their water consumption and body weight were measured. Water consumption of NaF and ATX+NaF groups approximated those of the control group (Figure 5). The bodyweight of rats was measured on days 1, 7, 14, 21, and 28 of treatment. The NaF-treated rats failed to gain normal body weight, as compared to the controls, and the ATX pretreatment group showed a reversal in body weight when compared to the experimental group (Figure 6).

*Effect of NaF exposure and ATX pretreatment on learning and memory level changes*

**Figure 7:**
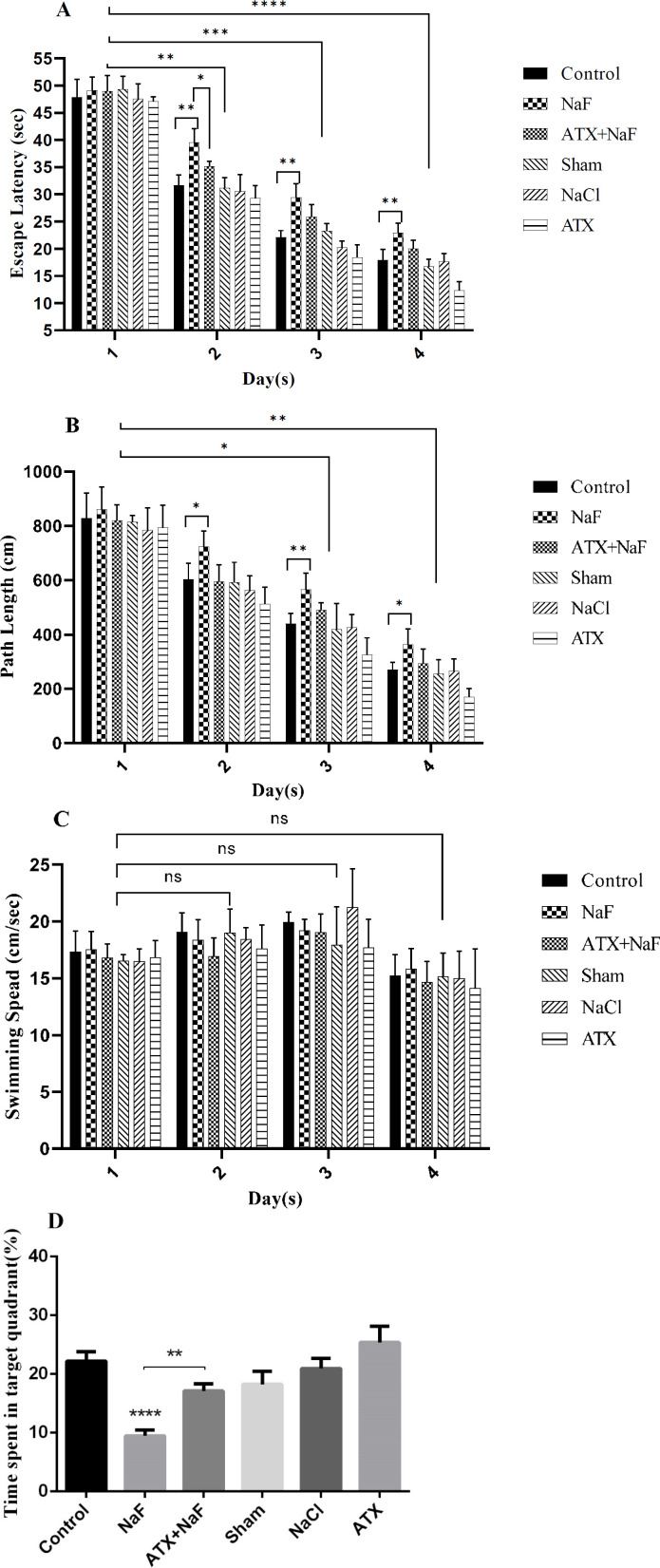
Effect of ATX administration on learning and memory changes induced by NaF using the MWM test. The mean value of path length (swimming distance), escape latency (time for finding a hidden platform), and swimming speed (velocity) during four continuous trial days in all treated and control groups are represented in A, B, and C, respectively. (D) The time spent in the target quadrant (%) was assessed by a probe test. The rats were treated with 270 ppm NaF alone (NaF), 25 mg/kg bw/day ATX pretreated (ATX+NaF), and 25 mg/kg bw/day ATX alone (ATX). The control group received no treatment, while sham and NaCl groups were treated with olive oil and NaCl solution, respectively

Animals were pretreated with ATX and subsequently treated with NaF, following which their learning and memory level changes were evaluated using an MWM test. As shown in Figure 7, the time needed to find the hidden platform and complete the swimming distance decreased during trial days in all groups, while the swimming speed did not change significantly during the experiment. However, the time spent finding the hidden platform and completing the swimming distance of rats exposed to NaF were significantly longer than those in the control group (*P* < 0.001, Figures 7A-7C). Pretreatment with ATX significantly improved escape latency and path length compared with the NaF-treated group (*P* < 0.001). NaCl and sham groups did not show a significant change in escape latency and path length compared with the control group. ATX decreased escape latency and path length compared with the control group (*P* < 0.001). These results show that exposure to NaF induces learning and memory impairment in rats, while ATX partially improves memory in rats. The probe test results showed a significant memory loss in the NaF group compared to the control group, whereas ATX administration significantly restores memory as compared with the NaF group (Figure 7D, *P* < 0.01).

*Effect of NaF exposure and ATX pretreatment on glutamate concentration in brain hippocampus*

Evidence suggests that excitatory amino acids (EAAs) can stimulate long-term potentiation (LTP), which has been considered the primary experimental model for investigating the synaptic basis of learning and memory (62). Glutamate is a powerful excitatory neurotransmitter in the brain, especially in the hippocampus, which is a critical region in the brain for learning and memory processes (63). It has been demonstrated that the duration of glutamate application can enhance LTP formation in the CNS (64). Therefore, to explore the toxic effects of NaF and neuroprotective potency of ATX on learning and memory, it would be worthwhile to determine the total concentration of glutamate changes in the hippocampus. 

**Figure 8:**
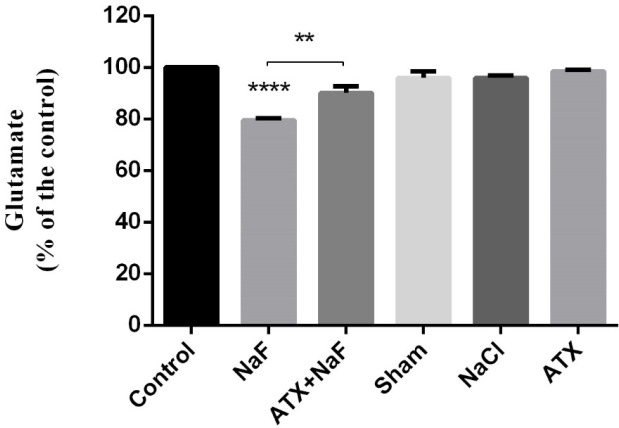
Effect of NaF exposure and ATX pretreatment on the glutamate concentration of rat hippocampus. The rats were treated with 270 ppm NaF alone (NaF), 25 mg/kg bw/day ATX pretreated (ATX+NaF), and 25 mg/kg bw/day ATX alone (ATX). The control group received no treatment, whereas sham and NaCl groups were treated with olive oil and NaCl solution, respectively

As shown in Figure 8, NaF significantly decreased glutamate concentration in the hippocampus of rats as compared to the control group (*P* < 0.0001). However, the glutamate level was increased in the ATX pretreatment group compared with the NaF group (*P* < 0.01). Glutamate level changes in the hippocampus of ATX, sham, and NaCl groups were not significant comparing with that of the control group. It seems that the deficiency and lower uptake of glutamate, as a neurotransmitter, have a key role in memory loss. 

Glutamate is the most plentiful amino acid in the diet. Nevertheless, little glutamate can cross the blood-brain barrier (65). Obviously enough, the fairly large quantities of glutamate through the extracellular space of the brain can raise the risk of depolarization of susceptible neurons and result in brain damage (66). As a result, a re-synthesis of glutamate holds in neurons as well as astrocytes. If an overall metabolic equilibrium is to retain in the brain, endogenous glutamate must synthesize to compensate for its loss. In the course of this process, AST and ALT have significant roles. Research indicates, for example, that the hippocampus AST and ALT activities are strongly inhibited in F- treated rats, while the activity of glutamate decarboxylase (GAD), which converts glutamate to GABA, increases (17, 67). This activation/inhibition causes a reduction in the total glutamate concentration in the rat hippocampus. Besides, it has been postulated that ATX returns to normal values the activity and levels of ALT and AST enzymes in blood and the liver (68, 69). Hence, it appears that the fluoride-mediated reduction in the total hippocampus glutamate concentration may be compensated by the ATX-induced activity of ALT/AST. 

*Effect of NaF exposure and ATX pretreatment on the production of intracellular ROS*

**Figure 9:**
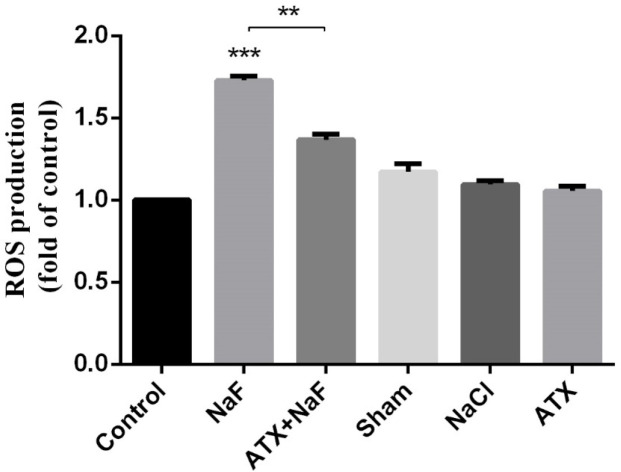
Effect of NaF exposure and ATX pretreatment on the production of ROS in rat hippocampus. The rats were treated with 270 ppm NaF alone (NaF), 25 mg/kg bw/day ATX pretreated (ATX+NaF), and 25 mg/kg bw/day ATX alone (ATX). The control group received no treatment, whereas sham and NaCl groups were treated with olive oil and NaCl solution, respectively

Intracellular ROS production was measured in the homogenate of the rat hippocampus. Similar to *in vitro* cell culture results, NaF caused a significant elevation in ROS production, whereas the pretreatment with ATX showed a remarkable suppression compared with the NaF group (*P* < 0.01). Also, ATX, sham, and NaCl groups did not show significant changes (Figure 9). 

*Effect of NaF exposure and ATX pretreatment on GLT1 protein expression*

**Figure 10:**
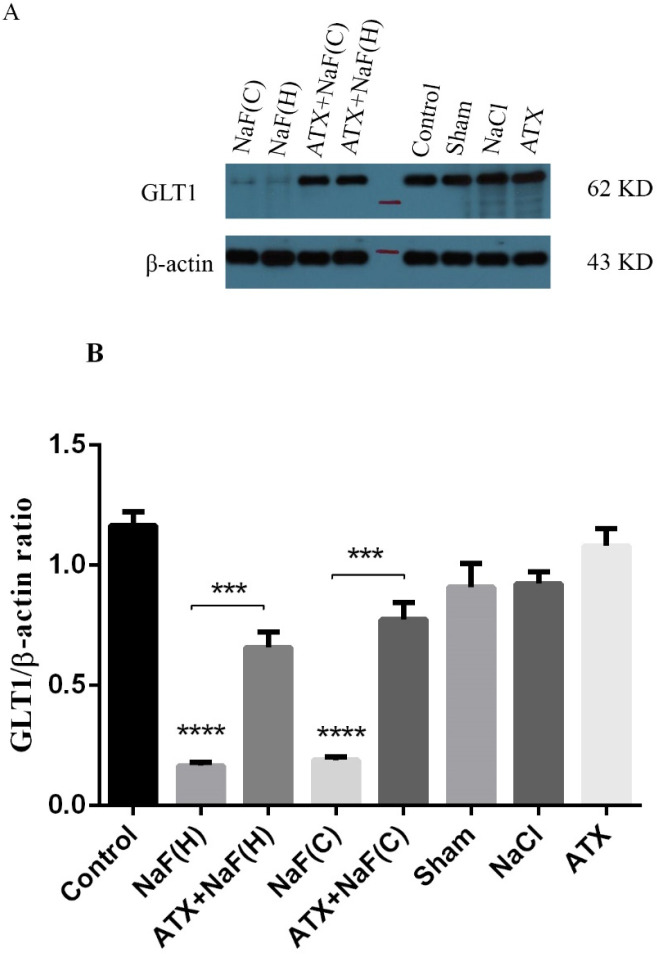
Effect of NaF exposure and ATX pretreatment on GLT1 protein expression in rat brain cortex (C) and hippocampus (H). (A) Western blot analysis of groups with exposure to 270 ppm NaF alone (NaF), 25 mg/kg bw/day ATX alone (ATX), and after pretreatment with 25 mg/kg bw/day ATX (ATX+NaF) was carried out after 24 h. The control group received no treatment, while sham and NaCl groups were treated with olive oil and NaCl solution, respectively. β-actin was used as a cell house-keeping protein. (B) The expression of GLT1 was semi-quantitated as a ratio to β-actin

The expression of rat glutamate transporter (GLT1), a protein homologous with human EAAT2, was determined using the western blot technique. As shown in Figure 10, the protein expression of the brain hippocampus in the NaF group was suppressed compared with that of the control group, while pretreatment of rats with ATX improved GLT1 expression (*P* < 0.001). Also, protein expression changes in the brain hippocampus in ATX, sham and NaCl groups were not significant. These results were in good agreement with the in vitro study, in which the expression of EAAT2 transporter in NaF treated cells significantly decreased, while the cells pretreated with ATX restored the expression of the transporter. These indicate that similar mechanisms are involved in both in vitro human cells and the hippocampus of rat brains. 

## Conclusion

It is concluded that fluoride therapy in school children may lead to impaired memory, possibly due to disruption in glutamate uptake or synthesis, which results from ROS-mediated energy metabolism imbalance. However, lipid mimic antioxidants, such as ATX, can reverse this process by scavenging fluoride-induced ROS and restoring cell (mitochondria) energy production ability.

## Conflicts of interest

The authors declare that there is no conflict of interest regarding the publication of this article.

## Authors’ contributions

FMG, MRA, and FM carried out the experiments. FMG and MRA wrote the manuscript with support from FM, MS, and SK. SM and SK planed and helped with animal experiments. MS and SK helped in the supervision of the project. GR supervised the project.
